# A *CHRNB1* frameshift mutation is associated with familial arthrogryposis multiplex congenita in Red dairy cattle

**DOI:** 10.1186/s12864-016-2832-x

**Published:** 2016-06-30

**Authors:** Jørgen S. Agerholm, Fintan J. McEvoy, Fiona Menzi, Vidhya Jagannathan, Cord Drögemüller

**Affiliations:** Department of Large Animal Sciences, Faculty of Health and Medical Sciences, University of Copenhagen, Dyrlægevej 68, Frederiksberg C, DK-1870 Denmark; Department of Veterinary Clinical and Animal Sciences, Faculty of Health and Medical Sciences, University of Copenhagen, Dyrlægevej 16, Frederiksberg C, DK-1870 Denmark; Institute of Genetics, Vetsuisse Faculty, University of Bern, Bremgartenstrasse 109a, Bern, 3001 Switzerland

**Keywords:** Cattle, Congenital, Malformation, Rare disease, Neuromuscular disorder, Myasthenic syndrome, Linkage mapping, Gene test

## Abstract

**Background:**

Bovine arthrogryposis multiplex congenita (AMC) is a syndromic term for a congenital condition characterized by multiple joint contractures. Rare inherited forms of bovine AMC have been reported in different breeds. For AMC in Angus cattle a causative genomic deletion encompassing the agrin (*AGRN*) gene, encoding an essential neural regulator that induces the aggregation of acetylcholine receptors (AChRs), is known. In 2015, three genetically related cases of generalized AMC affecting Red dairy calves were diagnosed in Denmark.

**Results:**

The family history of three affected calves suggested an autosomal recessive inheritance. Single nucleotide polymorphism (SNP) genotyping showed a single genomic region of extended homozygosity of 21.5 Mb on chromosome 19. Linkage analysis revealed a maximal parametric LOD score of 1.8 at this region. By whole genome re-sequencing of the three cases, two private homozygous non-synonymous variants were detected in the critical interval. Both variants, located in the myosin phosphatase Rho interacting protein (*MPRIP*) and the cholinergic receptor nicotinic beta 1 subunit gene (*CHRNB1*), were perfectly associated with the AMC phenotype. Previously described *CHRNB1* variants in humans lead to a congenital myasthenic syndrome with impaired neuromuscular transmission. The cattle variant represents a single base deletion in the first exon of *CHRNB1* (c.55delG) introducing a premature stop codon (p.Ala19Profs47*) in the second exon, truncating 96 % of the protein.

**Conclusions:**

This study provides the first phenotypically and genetically characterized example of a bovine AMC phenotype that represents an inherited neuromuscular disorder corresponding to human congenital myasthenic syndrome. The identified *CHRNB1* loss of function variant is predicted to have a deleterious effect on fetal AChR function, which could explain the lethal phenotype reported in this study. The identification of this candidate causative mutation thus widens the known phenotypic spectrum of *CHRNB1* mutations and enables selection against this pathogenic variant in Red dairy cattle.

**Electronic supplementary material:**

The online version of this article (doi:10.1186/s12864-016-2832-x) contains supplementary material, which is available to authorized users.

## Background

Bovine arthrogryposis multiplex congenita (AMC) is a syndromic term for a congenital condition characterized by arthrogryposis (from Greek for ‘curved or hooked joints’) or joint contracture of multiple joints involving more than just one part of the body [1]. Most likely autosomal recessive inherited forms of bovine AMC associated with cleft palate, scoliosis and kyphosis have been reported in different cattle breeds such as Hereford [2] and Charolais [3, 4]. More recently, in American Angus cattle an outbreak of a recessive inherited form of AMC was reported to be associated with a 23 kb sized genomic deletion encompassing the entire ISG15 ubiquitin-like modifier (*ISG15*) gene, the 5’ regulatory region of the hairy and enhancer split 4 (*HES4*) gene, and the first two exons of the agrin (*AGRN*) gene (OMIA 001465-9913) (Additional file 1) [5]. In addition to these conditions, AMC occurs as an accompanying lesion of complex congenital syndromes [6] such as the bovine arachnomelia syndrome (OMIA 000059-9913, OMIA 001541-9913) [7, 8] and schistosoma reflexum [9].

Based on the wide spectrum of conditions associated with AMC in humans i.e. associated with more than 400 specific conditions [10], many although yet unrecognized causes of bovine AMC are expected to occur. Human AMC was initially defined as non-progressive congenital contractures that generally result from lack of fetal movement *in utero* and is usually considered generalized thus involving joints of both the spine and the limbs [1, 11]. Contractures are defined as joints that have reduced range of motion due to stiffening of normally flexible tissues [10]. Proper central and peripheral nervous system development and function are required for stimulation of muscle function. Muscle tissue, joints, ligaments, tendons, and skin require movement for normal development and function, without which, joints develop contractures. Reduced fetal movement *in utero* due to myopathic processes, motor neuron degeneration, vascular compromise, and abnormal skeletal or connective tissue development, limited space in the uterus, maternal illness, or toxin exposure can lead to multiple congenital contractures [10]. Therefore AMC is not a specific diagnosis, but rather a descriptive term as it may be caused by a wide range of genetic mutations as well as by non-genetic factors [10, 11]. In cattle, fetal exposure to Schmallenberg virus (SBV) may lead to loss of neurons in the brain and spinal cord, including spinal ventral horn motor neurons. This causes an imbalance in fetal muscular activity (flexor vs. extensor muscles), which is displayed as AMC at birth [12, 13].

In 2015, three stillborn Red dairy calves showing AMC were reported to the Danish surveillance program for genetic diseases in cattle [14]. This study reports detailed phenotypic and molecular investigations, which were performed to identify the likely genetic cause for this lethal defect.

## Methods

### Animals

Three Red dairy calves were submitted for examination: Case 1: a male delivered at gestation day (GD) 284; Case 2: a female delivered at GD 275; and Case 3: a female delivered at GD 258. The three cases originated from different herds. Two cases (Cases 1 and 2) were sired by the same artificial insemination bull. Initially, ethylenediaminetetraacetic acid (EDTA) stabilized blood samples were obtained from all three dams and semen samples were collected of both bulls. Later on, semen samples of three ancestral sires were obtained.

### Post mortem examinations

Initially full body computed tomography (CT) scanning using a single slice helical CT machine (Emotion, Siemens, Erlangen, Germany) with a slice thickness of 3 mm was performed to obtain a full view of the bone malformations. The calves were then necropsied. Specimens of brain, lung and spleen for virology and pleural effusion for serology were sampled in separate plastic containers and stored at -20 °C until analysis. Specimens of heart, lung, liver, kidney, spleen, adrenal gland, thymus, skeletal muscle, brain and spinal cord were fixed in 10 % neutral buffered formalin for histology and then processed by routine methods, embedded in paraffin, sectioned at 2–3 μm and finally stained with hematoxylin and eosin.

### Virology and serology

The herds were declared free from bovine virus diarrhea virus (BVDV) based on regular bulk tank milk analyses as part of the national BVDV surveillance. Fetal tissues were examined for SBV by real-time quantitative reverse transcription polymerase chain reaction (PCR) [15]. Examination for fetal and maternal antibodies against SBV was performed by an enzyme-linked immunosorbent assay (ELISA) (ID Screen® Schmallenberg Comp. ELISA) and for maternal antibodies against bluetongue virus (BTV) by an ELISA test kit (ID Screen® Bluetongue Competition). These analyses were performed at the National Veterinary Institute, Technical University of Denmark.

### Pedigree analysis

Five-generation pedigrees were constructed based on information obtained from the Danish Cattle Database and breeding associations and analyzed for inbreeding loops and shared ancestors. Initial investigation of pedigrees showed a close genetic relationship between the parents of all three cases.

### Genetic analysis

Genomic DNA was extracted from samples of eight animals (three cases and their respective dams and the two sires) and used for genotyping with the BovineHD BeadChip (illumina), including 777,962 evenly distributed SNPs, at Geneseek (Lincoln, NE, USA). Genomic DNA from additional three normal male ancestors was used for targeted genotyping of candidate variants during mutation analysis. To identify extended homozygous regions with allele sharing across cases, the following PLINK software commands were used: --maf 0, --max-maf 1.0, --geno 0.01, --hwe 0, --mind 0.15, --homozyg, --homozyg-match 1, --homozyg-group [16]. All given positions correspond to the bovine UMD3.1 genome assembly. Multipoint parametric linkage analyses were performed with MERLIN software version 1.1.2 [17]. For parametric linkage, LOD scores were calculated under the assumption of AMC segregating as a biallelic autosomal recessive trait, with complete penetrance. The frequency of the disease allele in the Red dairy cattle population is unknown and there is no data available that would make it possible to estimate the frequency in a reliable manner. For the calculations a frequency of 0.01 for the mutated allele was assumed.

Individual PCR-free fragment libraries with average insert sizes of 400 base pairs (bp) were prepared from the three affected calves which were sequenced to ~15x coverage on two lanes of an Illumina HiSeq3000 instrument using 2 x 150 bp paired-end reads. The mapping to the UMD 3.1 bovine reference genome assembly [18] and variant calling were undertaken as previously described [19]. The snpEFF software [20] together with the UMD 3.1 annotation was used to predict the functional effects of detected variants. We considered the following snpEFF categories of variants as non-synonymous: NON_SYNONYMOUS_CODING, CODON_DELETION, CODON_INSERTION, CODON_CHANGE_PLUS_CODON_DELETION, CODON_CHANGE_PLUS_CODON_INSERTION, FRAME_SHIFT, EXON_DELETED, START_GAINED, START_LOST, STOP_GAINED, STOP_LOST, SPLICE_SITE_ACCEPTOR, SPLICE_SITE_DONOR. The recent sequence variant database containing 1147 already sequenced genomes of the ongoing 1000 bull genomes project [21] was used as control cohort during filtering for private variants of the sequenced affected calves. In order to detect larger structural variants all annotated genes and loci in the candidate region were also manually inspected by visual control of the BAM files.

Genotyping of the two candidate variants for AMC was performed by re-sequencing a 247-bp PCR product using a forward primer (5-CCAATAACAGGTGCACATTCC-3) and a reverse primer (5-GCCTGGAGGAGGAAAGAACT-3) flanking the *CHRNB1* variant, and a 221-bp PCR product using a forward primer (5-GCACTGGTTTTTGCACATTC-3) and a reverse primer (5-TGTCTTTTTGCCTGCAGTTG-3) flanking the *MPRIP* variant. The PCR products were amplified with AmpliTaqGold360Mastermix (Life Technologies), and the products were directly sequenced using the PCR primers on an ABI 3730 capillary sequencer (Life Technologies). The sequence data were analyzed using Sequencher 5.1 (GeneCodes).

## Results

### Phenotype

**Fig. 1:**
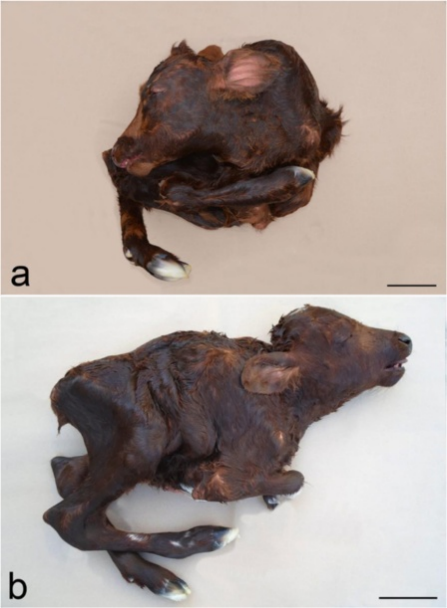
Gross morphology of arthrogryposis multiplex congenital (AMC). **a** and **b** Severe generalized contracture of joints of the spine and limbs in two calves. **a** The spine shows an almost 180° scoliosis, so that the head approached the caudal part of the calf. Case 2, body weight: 15.0 kg. **b** The neck is short due to cervical scoliosis and torticollis. The distal part of the front limbs were teared of during assisted vaginal delivery, Case 3, body weight: 11.0 kg. Bar = 10 cm

All cases had reduced body weight (15.2 kg, 15.0 kg and 11.0 kg, respectively, compared to normally around 39.6 kg for females and 44.1 kg for males) and displayed severe generalized contracture of joints of the spine and limbs with flexion of all joints, except the phalangeal joints that were extended. Vertebral lesions consisted of combinations of torticollis and kypho-scoliosis affecting the entire spine. Case 3 also had mild lordosis. Cases 1 and 2 had an almost 180° scoliosis of the thoraco-lumbar spine, so that the head approached the caudal part of the calf (Fig. 1). The joints had fibro-osseous ankylosis. All cases had palatoschisis, while cases 1 and 2 had slight lateral deviation of the viscerocranium. Case 2 also had deformation of the left side of the skull due to compression from a limb. The thorax and abdomen were narrowed thus compressing the organs and the ribs had an uneven course. All cases had generalized severe lipomatous muscular atrophy.

Internal organ lesions were associated with the narrowing of the body cavities. The apex of the heart was abnormally pointed and cases 2 and 3 had dilation and hypertrophy of the right ventricle. The lung was compressed and had diffuse congenital atelectasis. Additional lesions consisted of abdominal bilateral cryptorchidism (case 1), dilated urinary bladder with diverticulae (case 2) and edema and a subcutaneous cyst in the ventral neck (case 3). The brain and spinal cord appeared grossly normal.

**Fig. 2:**
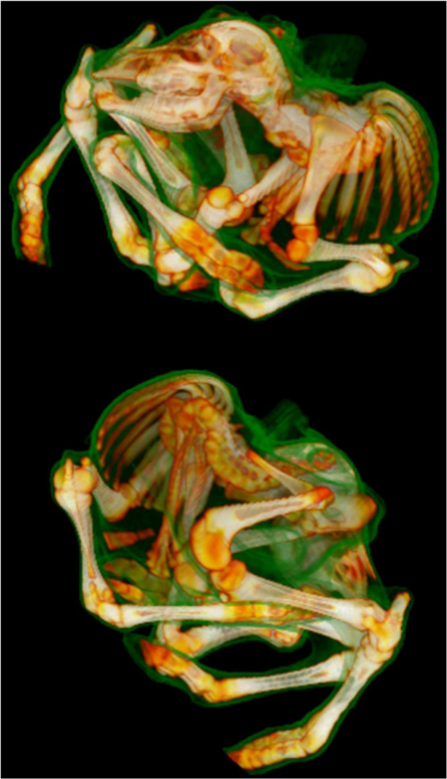
Surface rendered computed tomography images in two projections of a case of arthrogryposis multiplex congenital (AMC). The scanning data are rendered with bone and soft tissue with the latter set to have a degree of transparency in the reconstruction thus allowing visualisation of the calf’s overall morphology and its relation to the underlying skeletal abnormalities. Images prepared using OsiriX: An open-source software for navigating in multidimensional DICOM images

CT scanning confirmed the skeletal lesions found at necropsy, but provided a more detailed morphology of skeleton, which was difficult to assess in detail due to the generalized ankylotic arthrogryposis. The fusion between adjacent vertebrae resulted in a rigid structure and deformities in the thoracic and lumbar vertebrae combined together formed a twisted, open helical shaped spine. The ribs were thickened. Fusion of the dorsal spinous processes was seen in all vertebral regions, while the size and shape of the lumbar transverse processes were asymmetrical. The pelvis was deformed, and attached asymmetrically to the sacral vertebrae (Fig. 2). A CT scan movie showing one of the cases (case 1) as an example is presented in Additional file 2. The spinal canal appeared patent throughout its entire length with no sites of compression and the cranial cavity was of normal size and shape.

**Fig. 3:**
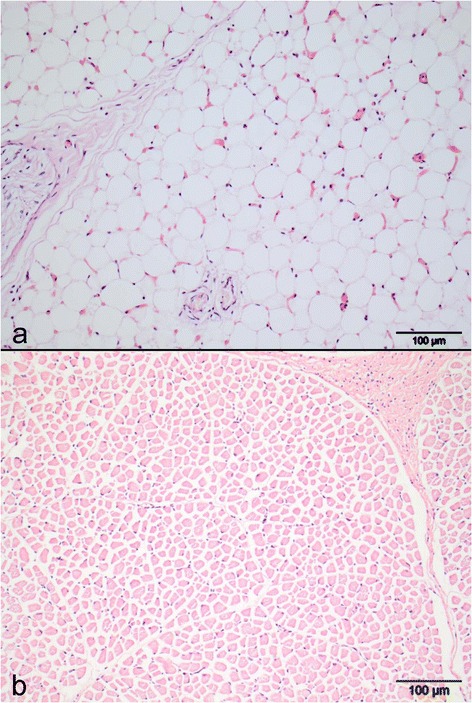
Photomicrophotograph of muscle lesions. **a** Severe lipomatous muscular atrophy with complete absence of normal muscle fibers; **b** Normal muscle of a newborn calf showing a normal muscle morphology (for comparison). Musculus semitendinosus, hematoxylin and eosin

Histology showed complete lipomatous muscular atrophy (Fig. 3) and congestion in several other tissues. Congestion was particular prominent in the lungs, which also had arterial muscular hypertrophy. Lesions were not observed in the central nervous system and the number of spinal ventral motor neurons appeared within normal range.

Examination for SBV and antibodies against SBV and BTV were negative.

### Genetic mapping of the causative mutation

**Fig. 4:**
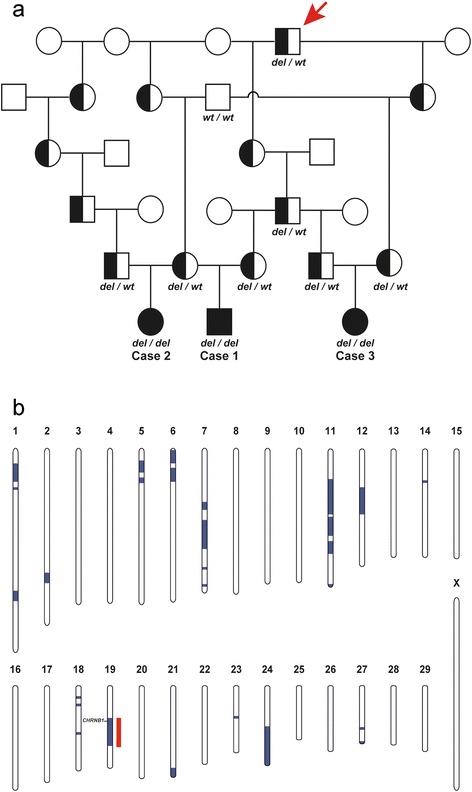
Recessively inherited arthrogryposis multiplex congenital (AMC) in Red dairy cattle maps to chromosome 19. **a** Familial relationships between the three AMC affected calves (filled symbols). Males are represented by squares, females by circles. Half-filled symbols represent healthy obligate heterozygous carriers and open symbols represent healthy relatives with an unknown genotype. DNA samples were available only from 11 animals of which the CHRNB1 genotypes are shown below the symbol. The common male ancestor (The sire Peterslundborn in 1997) and possible founder animal is marked by a red arrow. **b** Parametric linkage analysis for a recessive trait in the family and homozygosity analysis across the three AMC cases yielded several linked genome segments (*blue*) and a single homozygous genome segment (*red*). Only one region on chromosomes 19 showed both linkage and homozygosity and were considered as the critical interval (the position of the AMC associated *CHRNB1* gene is indicated)

**Fig. 5:**
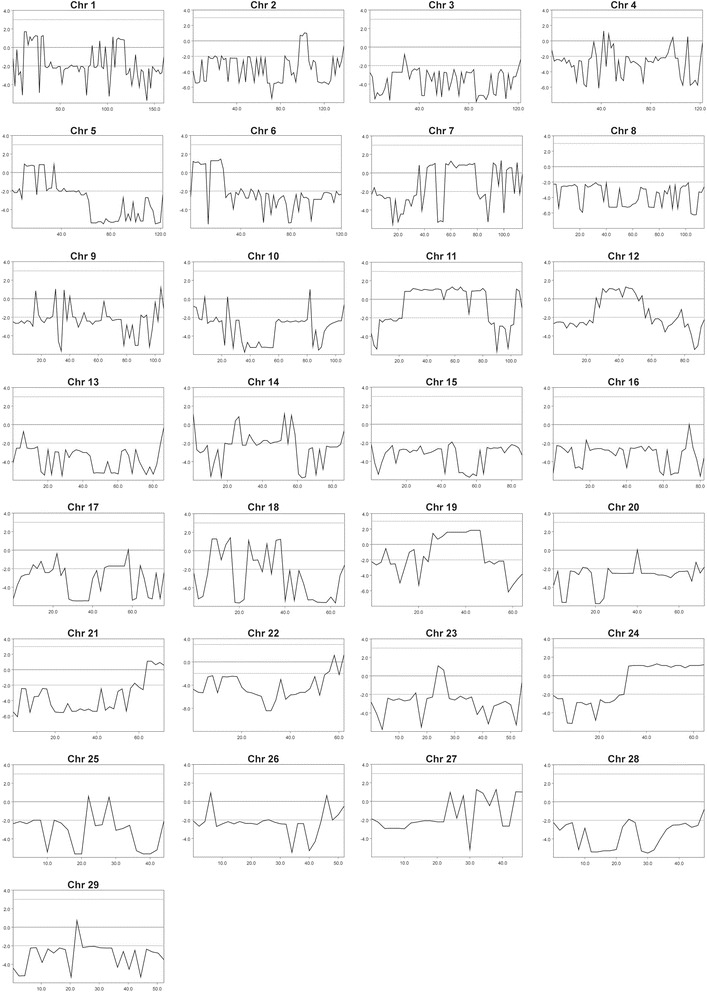
Parametric linkage analysis assuming monogenic recessive inheritance. The estimated multipoint LOD score (y axis) at a particular location (x axis) along a grid of 2 Mb equally spaced locations of all 29 autosomes. Note the positive results on chromosomes 19 with maximum LOD scores of 1.83 for a 20 Mb region suggested linkage of arthrogryposis multiplex congenita (AMC)

The pedigree of the established Red dairy cattle family segregating for AMC was consistent with monogenic autosomal recessive inheritance (Fig. 4a). In all three AMC cases, the parents were healthy and all affected calves could be traced back to a single common male ancestor born in 1997, as their parents had this bull as common ancestor 3 or 5 generations ago, respectively. Under this scenario, the AMC affected calves were considered to be identical by descent (IBD) for the causative mutation and flanking chromosomal segments. A homozygosity mapping approach was therefore applied to determine the position of the mutation in the bovine genome. The three cases were analyzed for extended regions of homozygosity with simultaneous allele sharing using genotypes of more than 770,000 evenly spaced SNPs. A single genomic region on cattle chromosome 19, in a region containing 3619 SNP markers corresponding to a 21.54 Mb interval from 26.51 to 48.05 Mb, was identified being IBD in the genotyped cases (Fig. 4b). Genotyping data of 532,965 polymorphic SNP markers of eight available animals were used for linkage analysis in the presented Red dairy cattle family segregating for AMC. The estimated maximal parametric LOD score of 1.83 for a 20 Mb region suggested linkage of AMC to the IBD region identified before (Fig. 5). Taken together this genome interval on chromosome 19 was considered the minimal critical interval for the subsequent analyses.

### Mutation identification

To obtain a comprehensive overview of all variants in the critical interval, the whole genome of all three available AMC cases were re-sequenced. SNPs and indel variants were called with respect to the reference genome of a presumably non-affected Hereford cow. Due to the recessive inheritance and the lethal effect of the mutation we hypothesized that most likely a non-synonymous loss of function mutation affecting the coding sequence of a gene would be responsible for AMC. Therefore subsequently only variants that were homozygous in the affected calves are reported. The visual inspection of the BAM files of the three sequenced cases revealed no evidence for the homozygous presence of a larger structural variant affecting coding regions of the critical interval on chromosome 19.

**Fig. 6:**
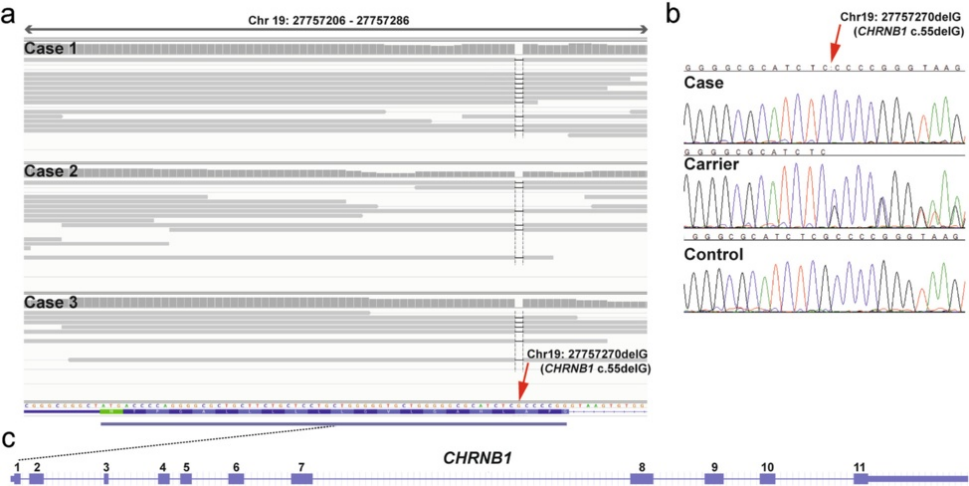
A *CHRNB1* frameshift mutation is associated with arthrogryposis multiplex congenita (AMC). A single G deletion, which was homozygous in all analysed cases, is marked by a red arrow. **a** IGV screenshot of the sequence alignments of three affected calves are displayed. Read pairs mapping to chromosome 19 are displayed in grey. Note the dashed black vertical line indicating the genome position of the G deletion. **b** Electropherograms of an AMC affected calf (case), a heterozygous carrier animal and normal control are shown. **c** The schematic representation of the bovine *CHRNB1* gene indicates that the 1 bp deletion is located in the first exon

Within the critical interval on chromosome 19 there were a total 17,342 homozygous variants including 385 coding variants within annotated genes, of which 138 were predicted to be non-synonymous. Subsequently, our membership in the 1000 bull genomes project was made use of [21] and the run4 variant database including 1147 genomes was used. It was hypothesized that the mutant allele at the causative variant should be completely absent from all other breeds outside Red dairy cattle as the pedigree analysis and the large size of the IBD haplotype clearly indicated a relatively young origin of the mutation. Therefore, excluding the genomes of 31 Red dairy cattle, a total of 1116 out of 1147 genomes were used from the 1000 bull genomes run4 variant database. This filter step allowed the exclusion of 16,905 non-coding and 383 coding variants remaining with 52 private variants located in intergenic and intronic regions and 2 private non-synonymous sequence variants that were absent from the other sequenced cattle of 29 different cattle breeds: Chr19:27757270CG > C or *CHRNB1* c.55delG and Chr19 35573959C > T or *MPRIP* c.2030G > A. Both coding variants were confirmed by Sanger sequencing and genotyped in the parents of the affected calves and additional three family members (Fig. 6). The genotypes showed perfect co-segregation with the AMC phenotype (Fig. 4b). In addition, both coding variants were absent in the genomes of the 31 Red dairy cattle bulls from Denmark and Sweden sequenced within the the1000 bull genomes project indicating that the associated haplotype on chromosome 19 containing the two coding variants is obviously rare.

## Discussion

Although the severity and anatomical location of the arthrogryposis varied slightly in the affected calves, they all suffered from widespread contracture of joints of the spine and limbs thus indicating a common pathogenesis. This was further supported by their genetic relationship, the presence of a common ancestor (Fig. 4a) and no traces of fetal exposure to the teratogenic SBV, which can produce a similar phenotype in susceptible fetuses. Based on these findings, a hitherto unrecognized genetic syndrome in Red dairy cattle was suspected. The extended disease associated IBD region of more than 20 Mb suggests that the recessive AMC causing mutation occurred quite recently. Therefore we assume that the distribution within the breed is probably restricted to the progeny of to the potential founder sire, which was born in 1997. However, within the recent years the founder sire has been used extensively for breeding in many countries due to his high genetic merit.

So far a genetic cause for bovine AMC has been reported only in Angus cattle (Additional file 1) [5]. Interestingly, besides two other genes, the described recessively inherited genomic deletion affects the *AGRN* gene. This gene encodes the protein agrin, which was originally identified as an essential neural regulator that induces the aggregation of acetylcholine receptors (AChRs) and other postsynaptic proteins on muscle fibers and is crucial for the formation and maintenance of the neuromuscular junction (OMIM 103320). Acetylcholine receptor expression at the motoric end-plates is not noticeably reduced in human agrin deficiency, a rare form of human congenital myasthenic syndrome (CMS) [22].

The AChR controls electrical signalling between nerve and muscle cells by opening and closing a gate, membrane-spanning pore to trigger muscle contraction. It has five subunits of four different types: two alpha and one each of beta, gamma (or epsilon), and delta subunits [23]. Mutations affecting subunits of the AChR pore cause CMS in humans (OMIM 100690). Generalized and fatigable skeletal muscle weakness is the most common clinical sign of CMS, but locus and allelic heterogeneity determine variable severity and additional symptoms. CMS can result from recessive missense, non-sense, or splice site and promoter region mutations in any of the AChR subunits, but most occur in the gamma (or epsilon) subunit [24]. The high frequency of mutations in the epsilon subunit compared with other subunits has been attributed to phenotypic rescue by substitution of the fetal gamma subunit for the defective epsilon subunit [25]. Individuals harboring null mutations in both alleles of *CHRNA1*, *CHRNB1*, or *CHRND* cannot survive because no substituting sub-units exist and hence these individuals probably die *in utero* [24]. Patients with heterozygous or homozygous low-expressor mutations in the non-epsilon subunit are severely affected have high mortality in infancy or early childhood [24]. Non-synonymous mutations in the human *CHRNB1* gene encoding the cholinergic receptor nicotinic beta 1 subunit are known to cause dominant and recessive forms of CMS type 2 (OMIM 616313 and OMIM 616314). Mice lacking AChR beta-subunit, the target of agrin, show abnormal neuromuscular synapse morphology [26].

In contrast, the *MPRIP* gene encodes myosin phosphatase Rho interacting protein, which is localized to actin myofilaments and binds to the myosin binding subunit of myosin phosphatase in vascular smooth muscle cells [27]. The MPRIP protein potentially plays a role in myosin phosphatase regulation [27] and is important for the progression of tumors [28]. Thus we concluded that the *CHRNB1*:c.55delG variant is much more likely to cause AMC in Red dairy cattle than the *MPRIP* missense variant (p.Arg677Gln). As the identified p.Ala19Profs47* premature stop codon is predicted to lead to a 96 % truncation of the bovine CHRNB1 protein, and the mutant mRNA is very likely targeted by the non-sense-mediated decay pathway, this bovine variant represents the most likely candidate causative mutation.

Here we demonstrate that an intensive surveillance programme identifying calves with similar disease phenotype in combination with the typical genetic structure of cattle populations and the availability of whole genome re-sequencing methods enables the identification of pathogenic variants causing inherited defects before a high number of diseased calves occurred. The identified genetic syndrome is associated with significant welfare issues and economic losses. If born alive, the syndrome is lethal to the offspring due to impaired respiration associated with musculoskeletal malformations or the offspring needs to be euthanized for welfare reasons. However, the generalized skeletal malformations may also cause dystocia requiring either assisted delivery or Caesarean section for the cow to survive. The magnitude of these issues may be significant for the Red dairy cattle breed unless preventive breeding measures are implemented as the common ancestor, which carries the mutation, has been used extensively in several countries.

The study therefore provides another example that the widespread use of elite sires by means of artificial insemination in livestock breeding leads to the frequent emergence of recessive genetic defects, which cause significant economic and animal welfare concerns [29].

## Conclusions

This study provides the first phenotypically and genetically characterized example that bovine familial AMC represents an inherited neuromuscular disorder corresponding to human CMS. Bovine familial AMC is confirmed to be associated with mutations in genes of importance for the neuromuscular junction. It is also the first reported *CHRNB1* loss of function mutation associated with a lethal neuromuscular phenotype in mammals. Finally, the findings provide a gene test to improve selection against this deleterious mutation in Red dairy cattle.

## Abbreviations

AChRs, acetylcholine receptors; *AGRN*, agrin gene; AMC, arthrogryposis multiplex congenita; BAM, binary version of a sequence alignment/map (SAM) file; Bp, base pairs; BTV, bluetongue virus; BVDV, bovine virus diarrhea virus; CMS, congenital myasthenic syndrome; CT, computed tomography; EDTA, ethylenediaminetetraacetic acid; GD, gestation day; *HES4*, hairy and enhancer split 4 gene; IBD, identical by descent; *ISG15*, ISG15 ubiquitin-like modifier gene; LOD, logarithm of the odds; PCR, polymerase chain reaction; SBV, Schmallenberg virus; SNP, single nucleotide polymorphism.

## Additional files

Additional file 1: Fact sheet on arthrogryposis multiplex from the American Angus Association. Downloaded from http://www.angus.org/pub/AM/AMFactSheet.pdf, 8 May 2015. (PDF 103 kb) Additional file 2: Surface rendered computed tomography images of a case of arthrogryposis multiplex congenita. (MOV 1951 kb)
